# Spinal Cord Stimulation: Mechanisms of Action, Indications, Types, Complications

**DOI:** 10.3390/jcm14238615

**Published:** 2025-12-04

**Authors:** Chrysoula Vlachou, Despoina Sarridou, Vasilios Grosomanidis, Ilias Voulgaris, Helena Argiriadou, Aikaterini Amaniti

**Affiliations:** 1 Faculty of Health Sciences, School of Medicine, Aristotle University of Thessaloniki, AHEPA Hospital, Stilponos Kyriakidi St., 546 36 Thessaloniki, Greece; chrysouv@auth.gr (C.V.);; 2 Department of Anaesthesia and Intensive Care, AHEPA University Hospital, 546 36 Thessaloniki, Greece; 3 Chronic Pain Unit, AHEPA University Hospital, Aristotle University of Thessaloniki, 546 36 Thessaloniki, Greece

**Keywords:** chronic pain, spinal neurosurgery, spinal cord stimulation, neuromodulation

## Abstract

Recent advances in neuromodulation are opening new pathways for treating chronic pain, with spinal cord stimulation (SCS) poised for substantial transformation in the coming years. Evolving technologies and a deeper understanding of pain mechanisms are driving a move away from traditional, standardized stimulation models toward more precise and personalized interventions. This shift reflects not only technical progress but also a growing emphasis on tailoring treatment to individual patient profiles. Advances in neuromodulation have introduced new stimulation patterns such as high-frequency, burst and dorsal root ganglion stimulation, which developed to address the limitations of conventional tonic SCS, especially declining long-term efficacy and the need for paresthesia. Early studies show promising results for these newer modalities, but findings are inconsistent and long-term data remain limited. In this article, we explore the current landscape of SCS innovation, highlight emerging clinical approaches and discuss the conceptual and technological trends that are likely to redefine the role of neuromodulation in chronic pain management.

## 1. Introduction

Pain is a complex, subjective experience that encompasses both sensory and emotional components and is typically linked to actual or potential tissue injury or is described in such terms. When pain persists beyond the expected period of tissue healing it is classified as chronic. Chronic pain represents a significant global health challenge, affecting hundreds of millions of individuals and imposing substantial economic burdens due to healthcare expenses and reduced productivity. Neuromodulation has emerged as a therapeutic strategy that is gaining widespread adoption for the management of chronic pain of diverse origins [1].

The clinical application of spinal cord electrical stimulation was first described in 1967 by Shealy et al., following the conceptual breakthrough of the Gate Control Theory introduced by Melzack and Wall in 1965. Since then, spinal cord stimulation (SCS) has evolved into a widely accepted intervention for managing refractory neuropathic pain and has been extended to various other chronic pain syndromes of neuropathic origin [2,3].

Neuropathic pain is a prevalent and often debilitating chronic pain condition as a result of injury or dysfunction within the somatosensory nervous system. It may arise directly from nerve damage or be secondary to metabolic disturbances, immune-mediated mechanisms, or inflammatory processes affecting peripheral nerves. Pharmacological treatments frequently offer only partial relief, mentioning the need for alternative therapeutic strategies. Spinal cord stimulation (SCS) has emerged as a valuable modality for managing various forms of neuropathic pain, including post herpetic neuralgia, persistent spinal pain syndrome, phantom limb pain, diabetic neuropathy and several types of cranial and peripheral neuralgias. By delivering electrical impulses to targeted regions of the spinal cord, SCS can modulate pain signaling pathways, leading to functional improvements and meaningful reductions in pain intensity for many patients [4].

This narrative review aims to provide a current overview of the fundamental physical principles and technical developments underlying clinical applications of spinal cord stimulation (SCS). With greater insight into the physiological mechanisms and continued refinement of the technological parameters of SCS, this knowledge may contribute to improved clinical outcomes and inform future research directions.

## 2. Methods

**Figure 1:**
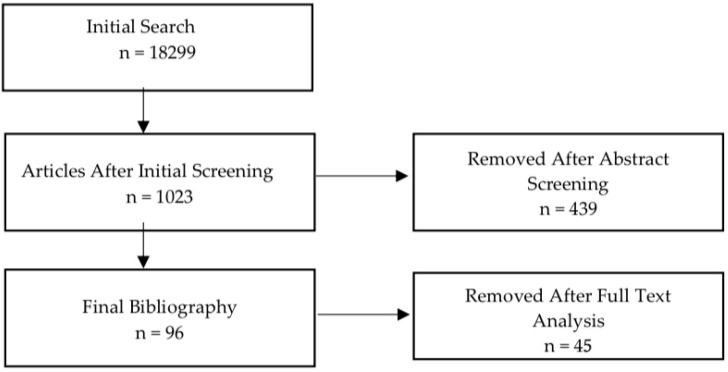
A flowchart describing the search process.

This narrative review was conducted using PubMed and Google Scholar to identify studies investigating the application of Spinal Cord Stimulation (SCS). The study selection process focused on research examining SCS in patients with chronic pain. The included studies were published from 2010 to September 2025. The literature search was conducted using the following search terms: “Spinal cord stimulation” AND “chronic pain” AND “neuromodulation.” The initial search results were imported into the Mendeley reference management system and duplicate records were removed. Titles and abstracts were then screened for relevance to the inclusion criteria referred to physiology, indications and contraindications, types of stimulation, and complications. Studies not published in English or that lacked relevance after abstract screening were excluded. Following this process, the final set of eligible studies was retained, forming the basis of the bibliography presented in Figure 1. Two reviewers independently screened titles/abstracts and performed full-text review; disagreements were resolved by consensus or consultation with a third reviewer. Findings related to this topic are characterized by methodological heterogeneity. Because the available studies display considerable methodological heterogeneity—including differences in study design, stimulation protocols, chronic pain etiologies, and outcome measures—we employed a narrative synthesis framework. This approach enabled us to contextualize diverse findings, highlight convergent trends, and identify areas where the evidence base remains inconsistent. Although this is a narrative review and not a systematic review, we assessed study quality and potential bias qualitatively. Selection bias was minimized by applying predefined inclusion and exclusion criteria to all identified studies, screening the literature in a structured manner and evaluating full texts independently to ensure that all eligible studies—not only those with positive findings—were included. More than one database was used to reduce the chance of selectively retrieving supportive evidence.

## 3. Gate Control Theory of Pain and Modern Mechanisms

Spinal cord stimulation delivers electrical impulses to the dorsal columns to modulate nociceptive transmission along ascending pain pathways. Although the gate control theory introduced by Melzack and Wall remains a foundational conceptual model, it represents only one component of a much more complex mechanistic landscape. According to this theory, activation of large-diameter Aβ fibers suppresses the transmission of nociceptive C-fiber input through segmental inhibitory interneurons within the dorsal horn [5]. Modern research indicates that SCS affects multiple levels of the neuraxis, including dorsal horn interneurons, supraspinal networks and neuroimmune circuits. The dorsal columns remain an optimal SCS target due to their anatomical segregation from motor pathways and their dense population of large-diameter afferents. Nevertheless, evidence from neurophysiology and functional imaging indicates that SCS modulates not only segmental gating but also descending inhibitory pathways, thalamocortical circuits and glial–immune interactions [5,6,7]. Furthermore, emerging evidence indicates that spinal cord stimulation exerts part of its analgesic action through attenuation of glial activation in the dorsal horn. In neuropathic pain models, spared nerve injury produces robust microglial and astrocytic upregulation—reflected by increased OX-42, GFAP and MCP-1 expression across superficial and deep laminae. Both low- and high-frequency SCS significantly suppress this reactive glial state, reducing microglial density and dampening astrocytic inflammatory signaling. By normalizing glial reactivity, SCS likely interrupts neuroimmune amplification of nociception and contributes to the reversal of central sensitization. These findings support a broader mechanistic framework in which SCS modulates not only neuronal circuits but also the glial networks that sustain chronic pain [7].

## 4. Spinal Cord Stimulation Physiology, Anatomy, Neurotransmitters and Mechanism

### 4.1. Spinal Segmental Physiology Mechanism

Specialized peripheral neurons transmit nociceptive information to the dorsal root ganglia and subsequently to the dorsal horn of the spinal cord, where they terminate in both superficial (laminae I/II) and deep (lamina V) layers. Within these laminae, nociceptive inputs undergo initial processing and modulation before being transmitted to supraspinal structures. The dorsal columns of the spinal cord carry large-diameter fibers that transmit precise sensory input and help modulate pain through gate control mechanisms. In contrast, small-diameter Aδ and C fibers ascend across the spinothalamic tract within the anterolateral column, conveying nociceptive signals to higher brain centers. The dorsal horn serves not only as a critical transmission center but also as an integrative hub for the processing and regulation of nociception to its transmission along ascending pain pathways [1].

### 4.2. Supraspinal Physiology Mechanism

Historically, C fibers were viewed primarily as merely channels for transmitting nociceptive action potentials in response to harmful stimuli. However, emerging evidence suggests that C fibers participate in more complex encoding mechanisms, such as marker line encoding, in which the frequency and pattern of action potential transition may dynamically vary in response to identical external stimuli. This functional plasticity contributes to enhanced neurotransmitter release and increased neuronal excitability, key features of peripheral and central sensitization [4]. Spinal cord stimulation modulates nociceptive signal transmission along the lateral spinothalamic tract, consequently affecting supraspinal structures such as the ventral posterior nucleus of the thalamus, somatosensory cortex, cingulate cortex and insular cortex [1]. This neuromodulatory effect may neutralize central sensitization—a process in which persistent nociceptive input, particularly across C fibers, increases the excitability of second-order neurons within the dorsal horn. Central sensitization is marked by long-lasting synaptic changes between C fibers and spinal interneurons and serves as a fundamental mechanism underlying the development and maintenance of chronic and pathological pain states [4].

### 4.3. Descending Pathways and Neurotransmitters

The descending inhibitory pain modulation system plays a crucial role in attenuating nociceptive signaling by transmitting information from supraspinal centers to the spinal cord, where it triggers inhibitory interneurons, which in turn diminish pain perception. This pathway emerges from several brain regions involved in pain modulation, including the periaqueductal gray, locus coeruleus, nucleus raphe magnus, rostral ventromedial medulla, anterior cingulate cortex, amygdala and hypothalamus. These descending signals travel through the brainstem—primarily through the medulla—and project to the dorsal horn of the spinal cord, where they modulate nociceptive transmission at the ‘spinal gate’. This modulation is mediated through serotonergic and noradrenergic pathways that influence neuronal activity within the dorsal horn [1,4]. As a result, key neurotransmitters mediating this descending inhibition include norepinephrine, serotonin (5-HT), dopamine and endogenous opioids. In individuals with neuropathic pain (NP), dysfunction within this regulatory system can result in an imbalance between excitatory and inhibitory inputs, contributing not only to persistent pain but also to comorbid symptoms such as anxiety, depression and sleep disturbances. Taking into account the above mentioned, impairment of descending inhibitory control is considered a central mechanism in the pathogenesis of chronic pain [4,8].

Experimental investigations into the mechanisms underlying SCS have demonstrated that it modulates neurotransmission within the dorsal horn of the spinal cord [2,9]. Evidence supports that neuropathic pain—characterized by peripheral hypersensitivity, allodynia and hyperalgesia—is primarily driven by central sensitization [2]. This process involves neurochemical alterations, notably an upregulation of excitatory neurotransmitters such as glutamate and aspartate, alongside a reduction in inhibitory gamma-aminobutyric acid (GABA)ergic tone, leading to elevated excitability of spinal nociceptive circuits [2,9].

Yadav et al., in a review focused on the application of SCS in Parkinson’s disease, proposed an additional mechanism of analgesia provided by SCS including restoration of the balance between oxygen supply and demand in ischemic conditions via attenuation of sympathetic nervous system activity [10]. Beyond the above mentioned, recent studies suggest, as additional mechanisms, that SCS may activate endogenous inhibitory pathways, possibly through the engagement of spinal progenitor cells, thereby contributing to the alleviation of neuropathic pain [4].

## 5. Indications and Contraindications

Spinal cord stimulation is a well-established treatment for chronic neuropathic pain, supported by a range of validated clinical indications. Its therapeutic efficacy depends on various factors, among which careful patient selection plays a significant role in the improvement of clinical efficacy.

Common clinical applications include persistent spinal pain syndrome (PSPS) without ongoing neurological deterioration [11], complex regional pain syndrome (CRPS) types I and II and nerve root-related pain [11,12]. SCS is also particularly beneficial in cases of axial low back pain, as well as in patients with upper extremity neuropathic syndromes, including radiculopathy [13,14].

Established indications, supported by randomized prospective studies, include persistent spinal pain syndrome (PSPS)—where contemporary trials demonstrate sustained improvements in pain and function [15]—complex regional pain syndrome (CRPS I/II) [13], refractory radiculopathy (including postsurgical radiculopathy) [16] and painful diabetic peripheral neuropathy (DPN) [15].

By contrast, emerging or investigational indications—such as nonsurgical axial low back pain [17] neuropathic upper-extremity syndromes, pelvic pain [18] and movement-related disorders such as gait dysfunction in Parkinson’s disease [19]—are supported mainly by small cohorts, pilot trials, or observational studies. These indications remain exploratory and the available evidence lacks long-term outcomes, adequate sample sizes and head-to-head comparative data. Explicitly distinguishing established from investigational uses is essential to avoid misrepresenting early-stage findings as mature clinical recommendations.

Additionally, SCS has been applied in selected cases of HIV-associated neuropathy and post herpetic neuralgia, though clinical outcomes can differ depending on the extent of deafferentation. Its efficacy has also been demonstrated in conditions such as peripheral nerve injury-related pain, intercostal neuralgia and phantom limb pain. These chronic pain syndromes are often marked by persistent and refractory symptoms, for which SCS represents an effective therapeutic option when conventional approaches have proven insufficient [11,20,21]. Spinal cord stimulation has shown promise in managing various forms of treatment-resistant neuropathic pain. It may offer significant relief in cases of painful diabetic peripheral neuropathy, particularly when pharmacological treatments are ineffective or poorly tolerated. In such cases, SCS has been shown to enhance limb salvage rates more effectively than surgical sympathectomy in non-reconstructable critical limb ischemia. In patients with chronic cancer-related pain, SCS should be reserved for those with a favorable prognosis, such as expected long-term remission or slowly progressing disease [14,15,16,17,18,19,20,21,22].

Other established indications include refractory angina pectoris unresponsive to optimal medical management, bypass surgery, or angioplasty and ischemic peripheral neuropathic pain due to peripheral artery disease—especially in patients with preserved microcirculation [15]. Overall, decisions regarding SCS indications must be tailored to the specific clinical pain pattern of each patient.

A scoping review by Sarah Flett et al. identified clinical studies indicating that SCS may support autonomic, homeostatic and metabolic functions related to movement and exercise. Lumbar SCS has been shown to normalize blood pressure in individuals with cardiovascular dysfunction due to high thoracic or cervical spinal cord injury (SCI) and to enhance temperature homeostasis in cases of impaired sweating. Additionally, it alters metabolic substrate utilization, which is associated with reduced perceived exertion during both lower limb movement and upper limb exercise [22].

The efficacy of lumbosacral SCS in restoring stepping, body position and even voluntary lower limb movement in individuals with motor complete SCI is widely recognized. SCS has also been reported to improve autonomic functions such as cough, sensation, bladder control, and intestine and immune regulation. Although the precise mechanisms remain unclear, evidence suggests shared neural structures and recruitment patterns between motor and autonomic circuitry, involving both direct and indirect activation of sympathetic pathways [23].

Physiological and anatomical changes during pregnancy can unmask non-obstetric pain conditions, whether preexisting or arising during gestation, significantly impairing quality of life. A systematic review by Camporeze et al. examined the use of SCS in pregnant patients, primarily for complex regional pain syndrome (CRPS) and persistent spinal pain syndrome (PSPS), which were among the most common etiologies reported. This review found that SCS was associated with notable success in pain reduction and a low complication rate, although complete pain relief was rarely achieved when SCS was used as a standalone therapy. Importantly, SCS demonstrated favorable cost-effectiveness compared to conventional pharmacological management strategies. Given its nonpharmacologic mechanism of action, SCS avoids many of the adverse effects, drug interactions and teratogenic risks associated with polypharmacy in pregnancy [24]. Meier et al., in a recent study presenting clinical data from a case series of six women treated with burst SCS during pregnancy, provide further support for the safety and feasibility of SCS in pregnant patients. Despite these promising findings, comprehensive clinical studies and large-scale cohorts are needed to better characterize the safety, efficacy and long-term outcomes of SCS in pregnant patients and to guide evidence-based use in this unique clinical context [24,25]. The main findings regarding the indications are summarized in Table 1.

While SCS offers significant therapeutic benefits in managing chronic neuropathic pain, careful consideration of contraindications is essential to ensure patient safety and optimize outcomes. Although spinal cord stimulation (SCS) can provide meaningful benefit for carefully selected patients with CRPS, several clinical situations represent clear contraindications to implantation. A major concern is the presence of persistent or unrecognized structural compression of neural elements. Residual mechanical pathology may be responsible for ongoing postoperative pain, and therefore a pre-implantation spinal MRI is essential. If imaging reveals a surgically correctable lesion, reoperation should be prioritized, as SCS is unlikely to offer benefit in this context. Thus, active compressive pathology constitutes an absolute contraindication.

Another important consideration is the accurate distinction between neuropathic and non-neuropathic pain. Despite the availability of validated tools—such as the Neuropathic Pain Questionnaire (NPQ), ID Pain, and PainDETECT—misclassification can occur. When the pain mechanism cannot be confidently identified as neuropathic, proceeding with SCS is inappropriate, making diagnostic uncertainty a relative contraindication [11].

Psychological status also plays a key role in treatment success. Many guidelines emphasize preoperative psychological screening to identify conditions that may interfere with outcomes. Severe psychiatric disorders, including major untreated depression, psychosis, or active substance abuse, are widely regarded as absolute or strong relative contraindications, as they can compromise postoperative adaptation and therapeutic response. Although the literature on depression and SCS outcomes remains mixed, the presence of unstable or severe psychiatric disease remains a critical exclusion factor [26].

Finally, the two-stage implantation process introduces additional considerations. The trial period, while useful for predicting treatment response, carries a notable risk of infection. Reported infection rates vary from 2.4% to 18.6%, influenced by surgical volume and operator expertise. Patients with high infection risk—due to immunosuppression, poor wound healing, or severe comorbidities—require careful evaluation, as these factors represent relative contraindications to SCS. Uncertainty remains regarding the predictive value of the trial phase, as randomized controlled evidence comparing direct implantation to trial-based approaches is lacking [11]. Table 2 summarizes the main findings related to the contraindications.

## 6. Types of Stimulation

### 6.1. Traditional Spinal Cord Stimulation

Conventional (tonic) spinal cord stimulation systems are capable of delivering electrical impulses across a broad frequency range, typically between 2 and 1200 Hz. Electrode placement is guided by the anatomical location of the patient’s pain. This pain is frequently accompanied by pronounced allodynia and cutaneous discoloration. To achieve optimal coverage of the painful area, electrodes are strategically implanted above the region of interest to engage and modulate ascending nociceptive pathways within the spinal cord and higher central structures [26,27].

### 6.2. Tonic Stimulation

Tonic spinal cord stimulation typically utilizes a pulse width ranging from 100 to 500 μs. This approach delivers continuous, low-frequency electrical pulses in a frequency between 30 and 100 Hz. Typically stimulation amplitude in the range of 40 to 60 Hz evokes paresthesia, perceived as tingling or burning sensations. The perception of paresthesia is individually based on the patient’s tolerance. Paresthesia perception arises predominantly from the activation of large Aβ dorsal column axons. This is particularly concerning in patients with severe allodynia, where the presence of such symptoms may diminish the therapeutic efficacy of SCS or lead to poor tolerability [26,28,29]. Optimal therapeutic outcomes are achieved when these paresthesias spatially correspond to the patient’s pain distribution, effectively masking the painful stimuli. Clinical studies have demonstrated that tonic SCS can significantly reduce pain in patients with persistent spinal pain syndrome (PSPS), as well as in those with complex regional pain syndrome (CRPS), highlighting its benefits in managing chronic neuropathic pain conditions [20,27,29].

### 6.3. High-Frequency Stimulation

Conventional spinal cord stimulation delivers electrical impulses at frequencies below 1200 Hz (conventionally commonly used in 60–200 Hz) [29] with the primary objective of masking pain perception by causing paresthesias that overlap the painful region. In contrast, high-frequency SCS operates at frequencies up to 10,000 kHz (HF10) and achieves analgesia without producing paresthesias, distinguishing it from tonic stimulation [20,23]. High-frequency SCS has become an established therapeutic option for managing neuropathic pain syndromes with diverse underlying causes. Its analgesic effects are thought to result from reduced sensitivity of hyperactive wide dynamic range (WDR) neurons and modulation of central sensitization processes, including suppression of the “wind-up” phenomenon [26].

Developed as part of the second generation of SCS technologies aimed at delivering paresthesia-free neuromodulation, HFS offers a therapeutic alternative for patients who are unable to tolerate the sensory experiences typically induced by tonic stimulation. Preclinical investigations have proposed several mechanisms underlying the effects of HFS, including inhibition of axonal conduction, modulation of glial–neuronal signaling and disruption of pathological neural activity [20].

Despite its expanding clinical use, the mechanistic basis of HF10 remains a topic of active debate. Early preclinical work suggested that high-frequency stimulation may produce a reversible conduction block of dorsal column axons; however, subsequent modeling and electrophysiological studies challenge this interpretation, noting that the stimulus amplitudes used clinically are insufficient to sustain a true conduction block. An alternative hypothesis proposes that HF10 exerts its analgesic effects through neuronal desynchronization within dorsal column pathways, disrupting pathologic burst firing and dampening central sensitization. These competing theories reflect the broader uncertainty regarding how HF10 achieves paresthesia-free analgesia and underscore the gap between clinical efficacy and mechanistic clarity. Integrating future neurophysiological and computational studies will be essential to resolve this controversy and refine patient-specific application of high-frequency stimulation [15,20].

### 6.4. Burst Stimulation

Burst spinal cord stimulation delivers stimulation in patterned pulses consisting of five spikes at 500 Hz, repeated at a frequency of 40 Hz, a configuration designed to approximate natural neuronal firing patterns [20]. Stimulation is applied at subthreshold amplitudes, which are sufficient to achieve analgesia without generating paresthesias. Burst SCS differs from conventional tonic stimulation both in its physiological effects and underlying mechanisms. Unlike tonic SCS, which activates paresthetic pathways and allows nociceptive signals, burst stimulation primarily modulates spinal dorsal horn activity and appears to act through non-GABAergic mechanisms [18,26,30]. Additionally, burst SCS does not induce paresthesias and may engage distinct neuroimmune processes. Remarkably, increased levels of the anti-inflammatory cytokine interleukin-10 (IL-10) have been observed in cerebrospinal fluid and systemic circulation following burst stimulation. IL-10 is thought to contribute not only to analgesia but also to the promotion of nerve fiber regeneration, suggesting a dual therapeutic role for burst SCS [26].

A growing body of evidence indicates that burst SCS provides analgesic outcomes comparable to those achieved with conventional tonic SCS, particularly in patients with persistent spinal pain syndrome (PSPS) or neuropathic radicular pain. Notably, patients seem to prefer burst stimulation, with improved pain relief frequently cited as a contributing factor. This preference has been shown to persist over long-term follow-up, with smaller crossover studies to have demonstrated that burst SCS results in more significant reductions in pain scores compared to both tonic stimulation and placebo, further supporting its clinical utility [20].

Emerging evidence suggests that burst spinal cord stimulation may exert its analgesic effects through modulation of supraspinal pain processing centers. Unlike tonic stimulation and placebo, burst SCS has been shown to enhance activity in cortical regions implicated in pain perception and cognitive-emotional processing. Electroencephalographic findings indicate increased activation in the dorsal anterior cingulate cortex and right dorsolateral prefrontal cortex following burst stimulation. Although these results may be influenced by concurrent interventions, they offer insight into potential central mechanisms of action [20]. Further research by De Ridder and colleagues demonstrated that burst SCS also increases activity in the pregenual anterior cingulate cortex, posterior cingulate cortex and parahippocampal region. This distributed activation pattern may facilitate modulation of descending inhibitory pathways and alter affective dimensions of pain, potentially explaining both the analgesic and mood-related benefits observed with burst SCS [31].

### 6.5. Dorsal Root Ganglion Stimulation

Dorsal root ganglion stimulation (DRGS) is a neuromodulation technique similar to SCS for managing both nociceptive and neuropathic pain, with a key distinction in its anatomical target. Rather than delivering stimulation to the dorsal columns, DRGS involves the placement of percutaneous leads over the dorsal root ganglion (DRG), which contains the cell bodies of primary sensory neurons [20,26].

One of the primary mechanisms underlying the analgesic effects of DRGS involves the selective activation of Aβ-, Aδ- and C-fibers through low-frequency stimulation. This targeted approach allows for the generation of action potentials at minimal frequencies, facilitating pain inhibition involving opioid receptor activation without significant contribution of the GABAergic system. Additionally, DRGS induces hyperpolarization of C-fiber membranes through calcium-activated potassium channels located in the T-junctions of primary sensory neurons. This hyperpolarization blocks the transportation of nociceptive signals toward the central nervous system, resulting in effective pain relief [26].

Functioning as a critical sensory gateway to the spinal cord, the DRG contains diverse populations of neurons that respond to mechanical, thermal, chemical and nociceptive stimuli. Given their role in sensory signal integration and modulation, the DRG has emerged as a compelling target for neuromodulation in the management of chronic pain. Therapeutic interventions directed at the DRG may modulate underlying pathophysiological mechanisms of neuropathic pain, particularly in cases where traditional dorsal column SCS is insufficient [32]. DRG stimulation has shown promise as an effective alternative for treating focal and refractory neuropathic pain syndromes, including postsurgical pain and phantom limb pain, due to its capacity for precise and segmental targeting of affected dermatomes [20,32].

### 6.6. DTM Spinal Cord Stimulation

Differential Target Multiplexed (DTM) spinal cord stimulation is a newer neuromodulation technique that targets not only neurons but also glial cells, which are thought to contribute to chronic pain. Unlike traditional tonic or high-frequency stimulation that mainly affects neuronal pathways, DTM delivers multiple synchronized electrical signals aimed at restoring balance between neurons and glial cells. This approach is supported by preclinical studies indicating that abnormalities in glial activity play an important role in maintaining neuropathic pain and central sensitization. Clinical evidence suggests that DTM can provide longer-lasting and more effective pain relief than conventional spinal cord stimulation, especially for patients suffering from chronic low back and leg pain. Controlled trials have shown that DTM increases the proportion of patients experiencing substantial pain reduction (≥50%), improves functional abilities and quality of life and may help reduce opioid use, all while maintaining a safety profile comparable to other SCS methods [33,34,35].

### 6.7. High-Density Spinal Cord Stimulation

High-Density (HD) Spinal Cord Stimulation is a newer technique that delivers a large number of electrical pulses at intensities below the level that normally causes tingling sensations. Compared to traditional SCS, this method increases both the frequency and total charge of pulses, which can help stimulate more nerve fibers and maintain effectiveness over time. Research shows that HD-SCS can help people with chronic neuropathic pain who did not get enough relief or experienced side effects from standard stimulation. For example, patients with conditions like failed back surgery syndrome or complex regional pain syndrome often report noticeable and long-lasting pain reduction for up to a year when switched to HD-SCS [36]. Other studies comparing subthreshold HD stimulation to conventional SCS found that HD often provided equal or better pain relief, was well tolerated, and was preferred by many patients. In practical clinical settings, carefully placing the leads and using sub-perception HD stimulation can produce effective, long-term pain relief, improving both daily functioning and sleep quality for patients who are not candidates for spine surgery [37]. Sub-perception HD stimulation is a type of spinal cord stimulation that reduces pain without the patient feeling any tingling or buzzing sensations (Table 3).

## 7. SCS and Complications

While spinal cord stimulation implantation is generally regarded as a safe procedure, a spectrum of acute and long-term complications has been reported. As shown in Table 4, these include infection, abscess formation, sepsis, incisional pain, cerebrospinal fluid (CSF) leakage, seroma, contact dermatitis, allergic reactions, thrombosis, wound dehiscence, hematoma or hemorrhage lead migration or fracture and electrode displacement [9,20,38,39,40,41].

Acute complications include the development of a neuraxial hematoma, which may occur during needle placement, lead advancement, or removal. Even a small epidural hematoma can produce rapid spinal cord or cauda equina compression, potentially resulting in irreversible neurological deficits or paralysis if not promptly recognized and surgically decompressed [16]. Subcutaneous hematoma in the pulse generator pocket or extension-lead pocket may also arise due to local tissue disruption during surgical preparation; however, these events typically remain extracanalicular and are more often managed conservatively [38,39,40].

Long-term and hardware-related complications are also a significant consideration. Lead migration or fracture remains the predominant long-term issue and a major driver of diminished therapeutic efficacy. Migration may shift paresthesia coverage or negate analgesia entirely, while lead fracture results in a complete loss of stimulation. These complications frequently necessitate surgical revision [23,41].

Most frequently, infections involve the pulse generator pocket or the dorsal incision. Although usually superficial, progression into the epidural space can occur and requires immediate intervention. Management strategies include targeted intravenous antibiotics for superficial infections and complete system explantation for deep or hardware-associated infections [16,23,42].

The most common medical complication is infection (3.8%), which is generally superficial. Pre-operative broad-spectrum antibiotics are typically used to minimize the risk of infection. There is no evidence that suggests routine post-operative antibiotic use is necessary [5].

Prevention strategies that have empirical support include strict perioperative asepsis, preoperative patient screening (to identify active infection, poorly controlled diabetes or other immunosuppressive states) and routine perioperative antibiotic prophylaxis [43]. Surgical techniques to reduce lead migration—secure anchoring, appropriate tunneling and limiting early postoperative axial loading—are recommended. Many centers emphasize activity restrictions during the initial weeks to promote fibrotic stabilization of leads [44]. For suspected deep infection or device-related epidural involvement, management commonly requires targeted IV antibiotics and device explantation when hardware is implicated [42]. Finally, rapid MRI and neurosurgical consultation are mandatory in cases suggestive of neuraxial hematoma to avoid permanent neurological injury [43].

## 8. Discussion

Chronic nonmalignant pain is a prevalent condition that creates a marked impact on both individual well-being and economic resources, with some studies estimating a global prevalence exceeding 30%. Spinal cord stimulation exerts its therapeutic effects through multiple mechanisms involving both spinal and supraspinal pathways, modulating various neurotransmitter systems [45]. Over the past decade, the field of neuromodulation has experienced rapid technological progress. Spinal cord stimulation has benefited from significant innovations in compatible devices, thus giving more prospects regarding its use in the future [11,46].

Spinal cord stimulation is an established therapy for chronic neuropathic pain, with efficacy strongly influenced by appropriate patient selection. Its efficacy has been demonstrated across a range of conditions with chronic pain representing a common feature. More recently, its potential role during pregnancy has gained attention, offering a key advantage as a non-pharmacological treatment option [24,25]. Moreover, Karolina Opovaa et al. mention that preliminary evidence from case reports and small observational studies indicates that SCS may hold therapeutic potential for managing gait disturbances. Included conditions that support this are freezing of gait and postural instability, both of which are highly disabling features of advanced Parkinson’s disease (PD) [47]. The literature on these emerging applications—during pregnancy or treatment of gait disturbances in Parkinson’s disease—remains fragmented, with early reports showing promise but lacking methodologically robust data. These variations mention the need for cautious interpretation of existing evidence and underscore the complexity of predicting therapeutic outcomes across diverse patient populations.

Although the therapeutic potential of SCS is well supported across multiple indications, the evidence base is not uniformly consistent. Several studies report strong analgesic outcomes, while others demonstrate only modest benefit or variability in patient response despite similar clinical profiles. Conventional tonic stimulation has been widely validated as a treatment modality; however, its long-term efficacy is often limited. Specifically, certain patient cohorts demonstrate only limited pain relief or experience a significant reduction in effectiveness over time. This decline suggests potential underlying issues such as the development of patient tolerance, suboptimal placement of leads, or inherent heterogeneity within the mechanisms of neuropathic pain itself. This challenge is evident with conventional spinal cord stimulation while it offers pain relief to approximately half of all patients treated. This approach is frequently hindered by the pervasive issue of long-term tolerance development, necessitating improved or alternative stimulation strategies [11,20].

This has driven the development of alternative stimulation patterns, such as burst and high-frequency stimulation, which are paresthesia-free and aim to improve outcomes in non-responders [11]. Despite the rapid technological progress in neuromodulation, considerable controversy persists regarding the relative effectiveness of the various SCS paradigms. While burst and high-frequency stimulation were developed to address the limitations of conventional tonic SCS—particularly long-term tolerance and the need for paresthesia—published outcomes remain inconsistent across studies. Some reports demonstrate superior pain relief and improved patient satisfaction with paresthesia-free waveforms, whereas others find no substantial difference compared with traditional stimulation.

While traditional tonic SCS (40–60 Hz) achieves analgesia through dorsal column activation and paresthesia-based coverage, clinical response is variable and long-term efficacy is limited by tolerance and stimulation-induced discomfort [15,20,29]. In contrast, HFS uses a 10 kHz waveform that appears to suppress large-diameter fiber activity (which normally produces paresthesias) while modulating smaller and medium-diameter afferents involved in pressure and vibration processing. This mechanism enables paresthesia-free analgesia [5]. In a pivotal randomized controlled trial, 84.5% of HF10 patients were responders for back pain and 83.1% for leg pain at 3 months, compared with 43.8% and 55.5%, respectively, in the tonic SCS group (*p* < 0.001). This corresponds to responder ratios of 1.9 for back pain and 1.5 for leg pain, with superiority maintained at 12 months. HF10 thus overcomes key shortcomings of tonic stimulation—namely suboptimal responder rates, positional variation and reliance on paresthesia [15].

Burst stimulation applies clusters of high-frequency pulses separated by quiescent intervals, achieving subthreshold neuronal effects with reduced charge delivery. By avoiding continuous tonic firing, burst SCS may also mitigate tolerance and tachyphylaxis that develop with long-term tonic stimulation [5]. In addition, burst stimulation has demonstrated superior analgesic efficacy compared with traditional tonic SCS across multiple pain domains. In comparative trials, burst stimulation reduced back, limb and overall pain intensity by 51%, 53%, and 55%, respectively, whereas tonic SCS achieved 30%, 52%, and 31% improvement. Burst also produced greater reductions in “pain now,” least pain, and worst pain (50%, 73%, and 36%) compared with tonic stimulation (26%, 46%, and 13%). Neurophysiological data further suggest mechanistic distinctions: burst stimulation preferentially activates the dorsal anterior cingulate cortex and right dorsolateral prefrontal cortex, regions implicated in affective-motivational pain processing, whereas tonic stimulation primarily modulates lateral sensory pathways. Despite these advantages, burst SCS is not universally superior; some patients respond equally well to tonic stimulation and optimal patient-selection criteria remain incompletely defined. Additionally, long-term durability and comparative cost-effectiveness data are still limited, underscoring the need for further head-to-head trials [31].

Beyond dorsal column targeting, neuromodulation of the dorsal root ganglion (DRG) and peripheral nervous system offers greater anatomical specificity [5,20,26]. The DRG—positioned at the junction of the peripheral and central nervous systems, containing primary sensory neuron cell bodies—serves as an effective site for modulating peripherally generated pain signals before they enter the spinal cord. The confined environment of the vertebral foramen provides mechanical stability for leads and minimizes positional variation, while the relative scarcity of cerebrospinal fluid reduces energy requirements and maximizes battery lifespan. DRG stimulation allows precise dermatomal targeting and is particularly advantageous for focal pain states, including refractory CRPS and localized neuralgias of regions poorly served by conventional SCS, such as the groin or foot [5]. Dorsal root ganglion stimulation (DRGS) has shown encouraging results for focal neuropathic pain such as CRPS, yet the magnitude and durability of these benefits vary considerably among trials. The anatomical precision of DRG targeting enables strong segmental specificity, yet this same anatomy introduces technical challenges: lead placement is more demanding and long-term lead stability can be problematic, particularly at mobile spinal levels. Reports of lead migration, increased energy requirements and position-dependent stimulation effects highlight the need for standardized implantation techniques.

Direct comparison of spinal cord stimulation modalities remains challenging due to several consistent methodological constraints within the literature, including small sample sizes, heterogeneous patient populations and inconsistent outcome measures across most available studies. While modalities such as tonic, high-frequency, burst, DRG, DTM and high-density stimulation all demonstrate analgesic potential, the evidence does not clearly establish the consistent superiority of any single approach across various indications. The literature presents varied, yet often encouraging, findings regarding the duration and durability of pain relief. For instance, pain relief achieved with conventional SCS was reportedly maintained over a mean follow-up period of 24 months [27]. Similarly, studies focusing on burst SCS have reported sustained pain relief for up to 6 months. However, reported advantages of high-frequency and burst stimulation—such as paresthesia-free analgesia—are not consistently replicated across all trials and long-term follow-up beyond these periods often remains limited [26].

Taken together, while newer paradigms offer meaningful advantages, the evidence base is fragmented, with inconsistent outcome measures and an absence of robust head-to-head trials to determine true comparative superiority. A broader limitation of the SCS literature is the substantial methodological heterogeneity across studies. Many published trials include small sample sizes, single-center cohorts, retrospective studies or open-label designs, reducing generalizability and increasing susceptibility to bias. Follow-up durations vary widely, making it difficult to draw firm conclusions regarding long-term durability of analgesia across stimulation modalities. Moreover, the field lacks adequately powered head-to-head trials comparing tonic, burst, high-frequency, DRG and newer approaches such as differential target multiplexed stimulation. Variability in outcome measures, patient selection criteria, device programming strategies, and reporting standards further complicates cross-study comparisons. These limitations should be acknowledged when interpreting existing findings and emphasize the need for harmonized methodologies in future neuromodulation research.

Spinal cord stimulation, while generally considered safe, is not without potential complications that can impact treatment efficacy and patient outcomes. These include both minor and major adverse events, such as allergic reactions, thrombosis, wound dehiscence, hematoma or hemorrhage, as well as mechanical issues like lead migration, electrode fracture or displacement. One significant complication is the formation of subcutaneous hematomas during the creation of implantation pockets for components such as lead extensions or the internal pulse generator. These events can compromise the stability and functionality of the system, ultimately affecting therapeutic success and leading to the need for careful perioperative management and follow-up [38].

Infection remains one of the most common complications of SCS, typically arising at the pulse-generator pocket or dorsal incision site and can be classified as device-related or superficial/deep surgical site infections, guiding appropriate management [5,39,48]. Superficial infections usually respond to targeted antibiotics, whereas deep or hardware-associated infections often require full system explantation [21,40,42]. Over the long term, hardware issues—particularly lead migration and lead fracture—remain the leading causes of diminished efficacy and frequently necessitate surgical revision [41]. Evidence-based prevention measures include strict aseptic technique, preoperative optimization of infection risks, routine perioperative antibiotic prophylaxis, secure lead anchoring and short-term postoperative activity restrictions [44,45].

## 9. Perspectives and Limitations

This review synthesizes current knowledge on spinal cord stimulation (SCS) and highlights emerging trends, technological advances and expanding clinical indications. Nonetheless, several limitations should be acknowledged. The included studies vary widely in design, methodology, and reporting standards, which limits direct comparability across findings. As this is not a systematic review, the selection of literature may introduce a degree of selection bias despite an extensive search of the available evidence. Evidence for newer approaches, including burst, high-frequency, and dorsal root ganglion stimulation, remains preliminary and long-term outcomes are not fully established. Additionally, while the review summarizes potential complications and contraindications, data on standardized risk stratification and patient selection criteria are limited. Despite these constraints, the review underscores the evolving landscape of SCS and emphasizes areas where future research, including larger multicenter trials and systematic outcome measures, could strengthen clinical understanding and optimize patient-specific therapeutic strategies.

## 10. Conclusions

This narrative review synthesizes current evidence on major SCS modalities—tonic, high-frequency, burst, DRG and DTM stimulation—and highlights their distinct mechanistic profiles, indications, contraindications and complications.

Spinal cord stimulation (SCS) has emerged as a valuable and evolving therapy for managing chronic pain, offering both efficacy and versatility across a range of conditions. Advances in technology have expanded its potential, while its non-pharmacological nature makes it particularly appealing in sensitive populations. However, successful outcomes depend on proper patient selection and meticulous management of potential complications. As the field continues to progress, SCS remains a promising component of modern pain management. Yet the persistent variability in patient response, the limited long-term durability of certain modalities, and the fragmented evidence surrounding emerging indications highlight the need for a more individualized and mechanistically informed approach. Future progress in neuromodulation is likely to be driven by closed-loop SCS systems, which use real-time physiologic feedback—such as local field potentials—to dynamically adjust stimulation and potentially reduce tolerance and non-responsiveness [49]. Parallel advances in personalized neuromodulation, leveraging patient-specific pain phenotypes, neurophysiological profiles and anatomically tailored targeting (including DRG and next-generation multiplexed stimulation), promise to refine therapy selection and improve outcomes. A major priority for upcoming research is the identification of biomarkers that differentiate responders from non-responders, enabling more precise patient selection and prediction of long-term benefit [50]. Achieving these goals will require larger, methodologically harmonized, head-to-head trials and consistent outcome measures to overcome the current heterogeneity of the literature and to guide SCS into a more evidence-driven, personalized therapeutic era.

## Figures and Tables

**Table 1:** SCS Indications.

Indication/Condition	Notes
Persistent spinal pain syndrome (PSPS) without neurological deterioration	Common indication; SCS effective if no progressive neuro deficits
Complex Regional Pain Syndrome (CRPS) Types I and II	Widely accepted application of SCS
Radicular and nerve root pain	SCS useful in nerve root syndromes
Axial low back pain	High-frequency SCS recommended in refractory cases
Non-reconstructable critical limb ischemia	Better limb salvage compared to sympathectomy
Painful diabetic peripheral neuropathy	Effective when medications are ineffective or poorly tolerated
Postherpetic neuralgia	Effectiveness may correlate inversely with deafferentation level
Peripheral nerve injury-related pain	SCS as an alternative in treatment-resistant pain
Intercostal neuralgia	Positive response reported in clinical use
Phantom limb pain	Common target for neuromodulation
Visceral pain (case-by-case)	Used selectively; individualized approach
Central neuropathic pain (segmental, SCI-related)	Localized segmental pain may respond to SCS
Chronic cancer-related pain (favorable prognosis)	Considered when disease progression is slow/remission likely
Autonomic/metabolic dysfunction (SCI-related)	Improves BP, thermoregulation, metabolism, and motor/autonomic functions

**Table 2:** SCS Contraindications.

Absolute Contraindications	Relative Contraindications
Persistent or surgically correctable spinal cord/nerve root compression identified on imaging	Diagnostic uncertainty regarding neuropathic vs. non-neuropathic pain mechanisms
Severe, uncontrolled psychiatric conditions (e.g., untreated major depression, psychosis)	Stable psychiatric conditions requiring further optimization before implantation
Active substance abuse or addiction	History of substance misuse currently under control
Active infection at surgical site or systemic infection	Increased infection risk due to immunosuppression, poor wound healing, or comorbidities
Inability to undergo required imaging or surgery	High perioperative risk associated with medical comorbidities

**Table 3:** SCS Modulated Pathways.

SCS Modality	Modulated Pathways/Mechanisms
Tonic Stimulation	Paresthesia-based mechanism primarily driven by activation of large Aβ dorsal column axons, resulting in downstream modulation of dorsal column–mediated sensory signaling.
High-Frequency Stimulation (HF10)	Produces analgesia through mechanisms that may include inhibition of axonal conduction, reversible conduction block of dorsal column axons and glial–neuronal signaling modulation. The precise mechanistic basis remains under active debate.
Burst Stimulation	Modulates spinal dorsal horn activity through non-GABAergic pathways; associated with increased IL-10 levels in CSF and systemic circulation (anti-inflammatory analgesia). Promoting nerve fiber regeneration. Modulation of supraspinal pain-processing centers.
Dorsal Root Ganglion Stimulation (DRGS)	Activation of Aβ-, Aδ-, and C-fibers. Enables pain inhibition through opioid receptor activation with minimal GABAergic involvement. Induces C-fiber membrane hyperpolarization via calcium-activated potassium channels in the T-junctions, blocking propagation of nociceptive signals into the CNS.
Differential Target Multiplexed (DTM) Stimulation	Delivers multiple synchronized electrical signals designed to restore homeostatic balance between neuronal and glial populations, targeting neuroimmune dysregulation implicated in chronic pain.

**Table 4:** SCS and Complications.

Complication Type	Specific Complications	Notes/Approximate Incidence Range
Infectious	Infection (pulse generator pocket, dorsal incision site) Epidural infection	Infections can spread into the epidural space; prompt management is critical. Incidence: 4.3%
Hemorrhagic	Neuraxial hematoma Subcutaneous hematoma Hemorrhage	Neuraxial hematoma may cause spinal cord compression or cauda equina syndrome; subcutaneous bleeding may occur during pocket formation. Incidence: 0.5%
Neurological	Dural puncture → CSF leak Traumatic neuraxial injury Spinal cord or cauda equina compression	Can result in serious complications such as irreversible deficits or paralysis
Mechanical/Device-related	Lead migration Lead fracture Electrode displacement	Lead migration alters paresthesia coverage; lead fracture may result in complete loss of stimulation. Incidence: 3.4%
Allergic/Immune	Contact dermatitis Allergic reaction	Rare, but possible response to implanted materials
Wound Healing	Seroma Wound dehiscence Incisional pain	Usually self-limiting; may delay recovery. Incidence: 0.4%
Vascular	Thrombosis	Rare but possible during implantation
Other	Adverse hardware reactions Discomfort at implantable pulse generator site	Hardware-related reactions are exceedingly rare; postoperative discomfort is typically transient.

## Data Availability

Not applicable.

## Abbreviations

The following abbreviations are used in this manuscript:

SCS	Spinal Cord Stimulation
PSPS	Spinal Pain Syndrome
CRPS	Complex Regional Pain Syndrome
SCI	Cervical Spinal Cord Injury
HFS	High-Frequency Stimulation
DRGS	Dorsal Root Ganglion Stimulation
DRG	Dorsal Root Ganglion
DTM	Differential Target Multiplexed
HD	High-Density
CSF	Cerebrospinal Fluid
